# Efficient and Provable Secure Pairing-Free Security-Mediated Identity-Based Identification Schemes

**DOI:** 10.1155/2014/170906

**Published:** 2014-05-26

**Authors:** Ji-Jian Chin, Syh-Yuan Tan, Swee-Huay Heng, Raphael C.-W. Phan

**Affiliations:** ^1^Faculty of Engineering, Multimedia University, 63100 Cyberjaya, Selangor, Malaysia; ^2^Faculty of Information Science and Technology, Multimedia University, Jalan Ayer Keroh Lama, 75450 Bukit Beruang, Melaka, Malaysia

## Abstract

Security-mediated cryptography was first introduced by Boneh et al. in 2001. The main motivation behind security-mediated cryptography was the capability to allow instant revocation of a user's secret key by necessitating the cooperation of a security mediator in any given transaction. Subsequently in 2003, Boneh et al. showed how to convert a RSA-based security-mediated encryption scheme from a traditional public key setting to an identity-based one, where certificates would no longer be required. Following these two pioneering papers, other cryptographic primitives that utilize a security-mediated approach began to surface. However, the security-mediated identity-based identification scheme (SM-IBI) was not introduced until Chin et al. in 2013 with a scheme built on bilinear pairings. In this paper, we improve on the efficiency results for SM-IBI schemes by proposing two schemes that are pairing-free and are based on well-studied complexity assumptions: the RSA and discrete logarithm assumptions.

## 1. Introduction

### 1.1. Background

Identification schemes allow one party, the prover, to prove itself to another party, the verifier, that it knows its secret key without revealing anything else about itself in the process. The main utilization of the identification primitive is to facilitate one-sided entity authentication and is conventionally deployed in access control mechanisms to facilitate resource control and distribution.

In traditional public key cryptography, certificates are used to ensure that a user's public key is legitimately bound to a particular user and cannot be replaced. This certificate is usually issued by a certificate authority. However, certificate management can become an issue when the number of users in a cryptosystem grows larger. One of the methods of mitigating this potentially costly problem is through the deployment of the cryptographic primitive in an identity-based setting introduced by Shamir [1].

Another issue that traditional public key cryptography faces is the revocation of user secret keys. This would necessarily involve the revocation of a user's certificate along with his public/secret key pair and is a costly operation. Timeliness is also a factor, as the procedure to revoke a user's keys may be a (relatively) long one and creates additional load on the certificate authority, turning the revocation procedure into a potentially costly process exercise. All these compound the certificate management issue mentioned earlier.

In the identity-based setting, where certificates are not used, key revocation is conventionally done by tagging validity periods onto the user's identity-string as an extension. This also creates an issue of timeliness since revocation is only possible at the end of those validity dates. Without checking certificates, it is also difficult to check if a user is still valid or if his user secret key has been revoked already.

In [2], Boneh et al. proposed the initial groundwork for instant revocation of user keys and privileges, including the public key and certificates by introducing the concept of security-mediated cryptography. The idea of security-mediated cryptography is to necessitate the cooperation of a security mediator, a trusted third party, in any form of transaction that a user needs to use. For example, in an encryption scheme, a security mediator needs to lend his cooperation to the user in order for the user to decrypt a particular ciphertext. And for signatures, a security mediator has to agree to cooperate with a signer in order to produce a valid signature.

Specifically, this is done by separating the user's secret key into two portions during the key generation. One portion of the key is given to the mediator while the other is given to the user. Therefore, the security mediator cooperates in the form of providing his portion of the secret key to be combined with the user's portion to create the full secret key for the transaction.

### 1.2. Related Work

Identification schemes were first introduced by Fiat and Shamir [3], while their identity-based counterparts, namely, identity-based identification schemes, were first formalized by Bellare et al. [4] and Kurosawa and Heng [5] independently. In recent years, there have been various advances in the area of identity-based identification, such as the introduction of identity-based identification schemes in the standard model [6, 7], hierarchical model [8–10], and certificateless model [11].

Following Boneh et al.'s initial work, Ding and Tsudik expanded on security-mediated cryptography to cover identity-based encryption using the RSA assumption [12]. Shortly thereafter, Libert and Quisquater proposed the first pairing-based security-mediated identity-based encryption schemes [13]. On the signature front, Cheng et al. proposed the first security-mediated identity-based signature [14].

Chow et al. [15] and Yap et al. [16] both extended security-mediated cryptography into the certificateless setting, where the issue of key escrow was addressed. In certificateless cryptography, first proposed by Al-Riyami and Paterson [17], the key generation center only produces half of the user secret key. The user then produces the other half of the user secret key to be combined into the full user secret key, thus securing their key from even the key generation center. However, the cryptographic primitives in the certificateless setting introduce more operational cost to the scheme and are known to be difficult to be proven secure [18].

### 1.3. Motivations and Contribution

In 2013, Chin et al. first combined the notion of security-mediated cryptography with identifications in the identity-based setting to propose the first security-mediated identity-based identification (SM-IBI) scheme [19]. In the paper, the authors provided the first formal definitions for SM-IBIs and also proposed a concrete construction. The motivation of the authors was to allow fast revocation of keys for identification schemes in the identity-based setting.

For SM-IBI schemes, a security mediator is required to participate in the identification protocol in order for a user to authenticate himself to a verifier. The security mediator can then hold a revocation list to identify which users' secret keys have been revoked and refuse participation for those users.

However the first concrete scheme that was proposed by the authors was built based on bilinear pairings. The pairing operation is widely known by cryptographers to be a costly operation; thus, it would be beneficial to construct pairing-free alternatives to facilitate more efficient running of the cryptographic primitive.

In this paper, we propose two pairing-free SM-IBI constructs as faster alternatives to the pairing-based scheme proposed by Chin et al. Our two schemes are constructed based on the RSA assumption and the discrete logarithm assumption, respectively. The RSA-based scheme, which we name as the GQ-SM-IBI, is constructed based on the Guillou Quisquater identification scheme constructed by Bellare and Palacio [20]. On the other hand, the discrete logarithm-based scheme, which we name as the BNN-SM-IBI, is constructed based on the BNN identity-based identification scheme proposed by Bellare et al. [4]. We provide security analysis for both schemes, proving them secure against impersonation under passive attacks if the RSA assumption and the discrete logarithm assumption hold and secure against impersonation under active and concurrent attacks if the one-more RSA inversion assumption and the one-more discrete logarithm assumption hold. Lastly, we provide an efficiency analysis, both theoretically and practically, and show that both schemes are significantly faster than Chin et al.'s pairing-based SM-IBI scheme.

The rest of the paper is organized as follows. We begin with some preliminaries and review the formal definitions and security model of SM-IBI schemes in Section 2. Then we show the construction and security analysis for the GQ-SM-IBI scheme in Section 3. This is followed by the construction and security analysis for the BNN-SM-IBI scheme in Section 4. In Section 5 we show the operational costs of both schemes as well as presenting our implementation results. Finally we conclude in Section 6.

## 2. Preliminaries

### 2.1. Discrete Logarithm Assumption

Let *G* be a cyclic group with prime order *q* and let *g* be a generator of *G*. The DL problem (DL) is defined as given a number *A* = *g* ^*a*^ in group *G*, output *a*.

**Definition 1 .**

The discrete logarithm assumption states that there exists no polynomial-time algorithm *M* that is able to (*t*_DL_, *ε*_DL_)-solve the discrete logarithm problem with nonnegligible probability such that

(1) $$\begin{array}{c} \text{Pr}\left[M\left(G,q,g,A\right)=a\right]\geq \varepsilon_{\text{DL}}. \end{array}$$

### 2.2. The One-More Discrete Logarithm Assumption

The one-more discrete logarithm (*OMDL*) problem was first introduced by Bellare and Palacio [20] in their proof against impersonation under active and concurrent attacks for the standard Schnorr identification scheme. Later work that involves proving security of identification schemes based on discrete logarithms to be secure against active and concurrent attacks for discrete logarithm also makes use of this assumption such as [4, 21].

Let *G* be a finite cyclic group of order *q* and let *g* be a generator of *G*. Define an experiment *E*_DL_ where an adversary *M* is given a challenge oracle *CHALL* that produces a random group element *W*_*i*_ ∈ *G* when queried and a discrete log oracle *DLOG*, which provides the discrete log *w*_*i*_ ∈ *Z*_*q*_ corresponding to the query *W*_*i*_ where *g* ^*w*_*i*_^ = *W*_*i*_. *M* wins if after making *i* queries to *CHALL*_DL_, *M* is able to output solutions to all *i* challenges with only *i* − 1 queries to *DLOG*, meaning *M* has to solve at least one instance of the discrete logarithm problem without relying on the discrete log oracle. *E*_DL_ returns 1 if *M* is successful and 0 otherwise.

**Definition 2 .**

The *OMDL* assumption states that there exists no polynomial-time algorithm *M* that is able to (*t*_*OMDL*_, *q*_*OMDL*_, *ε*_*OMDL*_)-solve the *OMDL* problem with nonnegligible probability where

(2) $$\begin{array}{c} \text{Pr}\left[E_{\text{DL}}(M\left(1^{k},G,g,{CHALL}_{\text{DL}},DLOG\right)=1\right]\geq \varepsilon_{OMDL}. \end{array}$$

### 2.3. RSA Inversion (RSAI) Assumption

Given $\left(N,e,X)\overset{\text{\$}}{\leftarrow }k_{RSA}(1^{k}\right)$ compute *Y* such that *Y* = *X* ^*d*^mod⁡*N* where *ed* = 1 mod⁡*ϕ*(*N*).

**Definition 3 .**

The *RSAI* assumption states that there exists no polynomial-time algorithm *M* that is able to (*t*_*RSAI*_, *ε*_*RSAI*_)-solve the *RSAI* problem with nonnegligible probability such that

(3) Pr[M(1k,N,e,X)=Y:Y=Xd  and  ed=1mod⁡ϕ(N)]≥εRSAI.

### 2.4. One-More RSA Inversion (OMRSAI) Assumption

This is the interactive variant of the *RSAI* problem first proposed by Bellare and Palacio [20] to prove security of the GQ identification scheme and is analogous to the *OMDL* assumption. This assumption is applied in the proof of security against active and concurrent attacks for RSA-based schemes.

Define an experiment *E*_*RSAI*_ where an adversary *M* is given $\left(N,e)\overset{\text{\$}}{\leftarrow }k_{RSA}(1^{k}\right)$ as input and access to two oracles *CHALL* and *RSA*. *CHALL* on any input returns a random point *W*_*i*_, while *RSA* on any input *h* will return *h* ^*d*^ where *ed* = 1  mod⁡*N*. *M* is required to compute the *RSAI* solutions to all the target points *W*_0_,…, *W*_*n*_ while using strictly less queries to the *RSA* oracle. In other words, *M* is required to find *W*_0_^*d*^,…, *W*_*n*_^*d*^ while using the *RSA* oracle only *i* < *n* times. *E*_*RSAI*_ returns 1 if *M* is successful and 0 otherwise.

**Definition 4 .**

The *O* *M* *RSAI* assumption states that there exists no polynomial-time algorithm *M* that is able to (*t*_*OMRSAI*_, *q*_*OMRSAI*_, *ε*_*OMRSAI*_)-win the *O* *M* *RSAI* problem with nonnegligible probability where

(4) $$\begin{array}{c} \text{Pr}\left[E_{RSAI}\left(M\left(1^{k},N,e,{CHALL}_{RSA},RSA\right)\right)=1\right]\geq \varepsilon_{RSAI}. \end{array}$$

### 2.5. Definition of Security-Mediated IBI Schemes

In this section, we review the definition of SM-IBI schemes as defined by [19]. The definition follows closely to that of conventional IBI schemes, with the difference being that the prover segment is extended to encompass obtaining tokens from the security mediator. The SM-IBI scheme is defined as five probabilistic polynomial-time algorithms.

- *Setup*. It takes in the security parameter 1^*k*^ as input and outputs the system parameters params  along with the master secret key MSK.
- *Extract.* Upon receiving a user's request for a key, it takes in params MSK and a user's identity ID. Once the secret key is created, the PKG separates the key into two portions, one for the user, *USK*_*user*_, and one for the security mediator, *USK*_*sem*_, and returns the portions to the respective parties.
- *Identification Protocol.* The identification protocol is an interactive protocol run by the 3 algorithms:* User-Prover* and* SEM-prover* on the prover side trying to authenticate himself to the* Verifier.* Both provers are used cooperatively in the interactive three-step canonical honest verifier zero knowledge proof of knowledge protocol with the verifier as follows.
  1. *User-Prover* initiates by sending his identity to ID to the* SEM-prover*.* SEM-prover* checks whether ID's keys have been revoked and stops with an error code if true. If ID is legitimate then it generates and sends SEM-COMMIT to User-Prover who then combines SEM-COMMIT with his own USER-COMMIT to form FULL-COMMIT to send to* Verifier.*
  2. *Verifier* selects a random CHALLENGE and sends it to the* User-Prover.*
  3. *User-Prover* relays CHALLENGE to* SEM-Prover* and receives SEM-RESPONSE from* SEM-Prover* which it then combines with his own USER-RESPONSE to form FULL-RESPONSE and sends to the* Verifier.* The* Verifier* will choose to either accept or reject it.

### 2.6. Security Model for Security-Mediated IBI

Adversaries of SM-IBI follow the description of standard identification schemes: passive and active/concurrent attackers. However, the adversary for SM-IBI is able to query additionally partial conversation components, specifically the user's prover and the security mediator's prover besides the usual full prover query.

The security of SM-IBI schemes is modelled as a game played by an adversary *I* against a challenger *C* as follows.

- *Setup. C* runs* Setup*, creates the system parameters params, and passes them to *I* while keeping the master secret key MSK to itself.
- *Phase 1.* This is the training phase. *I* is allowed to adaptively make the following queries to *C*.
  - *User-Extract *(ID). *C* will run* Extract* but returns only the user's portion of the secret key to *I*.
  - *SEM*-*Extract* (ID). *C* will run* Extract* but returns only the security mediator's portion of the secret key to *I*.
  - *Full-Extract *(ID). *C* will run* Extract* and returns both the user's portion of the secret key and the security mediator's portion of the secret key to *I*.
  - *Identification Queries *(ID). For passive adversaries, *C* will generate transcripts of valid conversations for *I*. For active/concurrent adversaries, *C* will act as the cheating prover, engaging *I* as the cheating verifier in conversations. *I* is able to issue any one of the following identification queries.
    - *SEM-Identification *(ID). *C* runs the security mediator's half of the prover session.
    - *User-Identification *(ID). *C* runs the user's half of the prover session.
    - *Full-Identification *(ID). *C* combines both security mediator's and user's session to generate a full and valid conversation.
- *Phase 2. I* will eventually output ID* on which it wants to be challenged on and begins its role as the cheating prover for both security mediator and user prover sessions. *C* on the other hand assumes the role of the verifier. *I* wins the game if it manages to convince *C* to accept with nonnegligible probability.

We say a security-mediated IBI scheme Π is (*t*_*SMIBI*_, *q*_*SMIBI*_, *ε*_*SMIBI*_)-secure under passive or active/concurrent attacks if for any passive or active/concurrent Type-1 impersonator *I* who runs in time *t*_*SMIBI*_, Pr[*I*  can  impersonate] < *ε*_*SMIBI*_, where *I* can make at most *q*_*SMIBI*_ full extract queries.

It is interesting to point out that extracting the security mediator's half of the secret key gives no information about the full user key and that neither security mediator's prover sessions nor user's prover sessions done alone will provide a valid conversation, but only the combined session will. This models the security requirement that any user cannot legitimately prove himself to a verifier without the security mediator's help.

## 3. GQ-SMIBI: RSA-Based Security-Mediated IBI Scheme

The GQ-SM-IBI scheme is derived from the GQ identification scheme proposed in [20] and is provably secure against passive attackers assuming the *RSAI* assumption and against active/concurrent attackers assuming the *O* *M* *RSAI* assumption.

The GQ-SM-IBI scheme is constructed as follows.

1. *Setup* (1^*k*^). It takes in the security parameter 1^*k*^, runs the key generation algorithm for RSA, and obtains an RSA instance, $\left(N,e,d)\overset{\text{\$}}{\leftarrow }k_{RSA}(1^{k}\right)$. It chooses a hash function *H* : {0,1}* → *ℤ*_N_* and publishes the system parameters *mpk* = 〈*N*, *e*, *H*〉. The master secret key *msk* = *d* is kept secret.
2. *Extract* (*mpk*, *msk*, *I*D). It takes in *mpk*, *msk* = *s*, and *I*D as input. It calculates *H*(*I*D) and sets *X*_*FULL*_ = *H*(*I*D)^*d*^. It further chooses $d_{SEM}\overset{\text{\$}}{\leftarrow }{\mathbb{Z}}_{\phi (N)}^{*}$, sets *d*_*USER*_ = *d* − *d*_*SEM*_, and calculates *X*_*USER*_ = *H*(*I*D)^*d*_*USER*_^ and *X*_*SEM*_ = *H*(*I*D)^*d*_*SEM*_^. It gives *X*_*USER*_ to the user as its partial secret key and *X*_*SEM*_ to the security mediator as its partial private key and keeps *X*_*FULL*_ secret.
3. *Identification Protocol.* It is run by the* User-Prover* and* SEM-Prover* with* Verifier* as such.
  - *User-Prover* sends its *I*D to* Sem-Prover* and chooses $y_{USER}\overset{\text{\$}}{\leftarrow }{\mathbb{Z}}_{N}^{*}$ and sets *Y*_*USER*_ = *y*_*USER*_^*e*^. SEM-Prover upon receiving *I*D checks if it is still valid and returns error if it is revoked. Otherwise,* SEM-Prover* chooses $y_{SEM}\overset{\text{\$}}{\leftarrow }{\mathbb{Z}}_{N}^{*}$ and sends *Y*_*SEM*_ = *y*_*SEM*_^*e*^ to* User-Prover*.* User-Prover* combines *Y*_*FULL*_ = *Y*_*SEM*_ × *Y*_*USER*_ and sends *Y*_*FULL*_ to* Verifier.*
  - *Verifier* chooses a random challenge $c\overset{\text{\$}}{\leftarrow }Z_{2^{l(k)}}$ where *l*(·) is a super-logarithmic challenge length such that 2^*l*(*k*)^ < *e*.* Verifier* then sends it to* User-Prover*.* User-Prover* relays *c* to* SEM-Prover*.
  - *SEM-Prover* calculates its half of the response *z*_*SEM*_ = *y*_*SEM*_ *X*_*SEM*_^*c*^ and sends it to* User-Prover*.* User-Prover* combines it with his response *z*_*USER*_ = *y*_*USER*_ *X*_*USER*_^*c*^ to create *z*_*FULL*_ = *z*_*SEM*_ × *z*_*USER*_ and sends it as a response to* Verifier*.

 Verifier checks if *z*_*FULL*_^*e*^ = *Y*_*FULL*_ *H*(*ID*)^*c*^ and accepts if yes; otherwise it outputs reject.

To check for completeness,

(5) ZFULLe=[(ySEMXSEMc)(yUSERXUSERc)]e=[ySEMyUSER(XSEMXUSER)c]e=ySEMeyUSERe(H(ID)(dSEM+dUSER)ec)=YSEMYUSER(H(ID)dec)=YFULL(H(ID)c).

### 3.1. Security Analysis: Impersonation under Passive Attack

**Theorem 5 .**

The GQ-SM-IBI scheme is (*t*_*SMIBI*_, *q*_*SMIBI*_, *ε*_*SMIBI*_)-secure against impersonation under passive attacks in the random oracle if the RSA Inversion Problem is (*t*_*RSAI*_, *ε*_*RSAI*_)-hard where

(6) $$\begin{array}{c} \varepsilon_{SMIBI}\leq \sqrt{\varepsilon_{RSAI}e\left(q_{SMIBI}+1\right)}+\frac{1}{2^{l(k)}}. \end{array}$$

**Proof**

Assume the GQ-SM-IBI scheme is (*t*_*SMIBI*_, *q*_*SMIBI*_, *ε*_*SMIBI*_)-breakable; then a simulator *M* that (*t*_*RSAI*_, *ε*_*RSAI*_)-breaks the *RSAI* problem can be shown. *M* takes in input (*N*, *e*, *y*) and runs the impersonator *I* as a subroutine. Without loss of generality, it can be assumed that any* SEM-Extract, User-Extract, SEM-Identification, User-Identification,* and* Full-Identification* queries are preceded by a* Create-User* query. To avoid collision and consistently respond to these queries, *M* maintains a list *L*_*H*_ = 〈*ID*_*i*_, *Q*_*ID*_*i*__, *f*_*ID*_*i*__, *X*_*FULL*_*ID*_*i*___, *X*_*SEM*_*ID*_*i*___, *X*_*USER*_*ID*_*i*___, *d*_*SEM*_*ID*_*i*___, *d*_*USER*_*ID*_*i*___〉 which is initially empty. The following shows how *M* simulates the environment and oracle queries for *I*.

(1) *Setup*. *M* selects a hash function *H* : {0,1}* → *ℤ*_N_* and sets the system parameters as *mpk* = 〈*N*, *e*, *H*〉. It sends *mpk* to *I*.
(2) *Create*-*User *(*ID*_*i*_). *M* chooses *l* ∈ {1,…, *q*_*H*_} randomly and lets *ID*_*l*_ = ID* at this point. Whenever *I* makes a *H* query on *ID*_*i*_, consider the following.
  (a) If *i* = *l*, *M* will choose $f_{{ID}^{*}}\overset{\text{\$}}{\leftarrow }{\mathbb{Z}}_{N}^{*}$ sets *Q*_ID*_ = *yf*_ID*_^e^ and will return *Q*_ID*_ to *I*. It adds 〈*ID*_*l*_, *Q*_ID*_, *f*_ID*_, *X*_*FULL*_ID*__ = ⊥, *X*_*USER*_ID*__ = ⊥, *X*_*SEM*_ID*__ = ⊥, *d*_*SEM*_ID*__ = ⊥, *d*_*USER*_ID*__ = *bot*〉 to *L*_*H*_.
  (b) Otherwise, for *i* ≠ *l*, *M* chooses $f_{{ID}_{i}}\overset{\text{\$}}{\leftarrow }{\mathbb{Z}}_{q}^{*}$, sets *Q*_*ID*_*i*__ = *f*_*ID*_*i*__^*e*^, and returns *Q*_*ID*_*i*__ to *I*. *M* also sets *X*_*FULL*_*ID*_*i*___ = *f*_*ID*_*i*__, chooses $d_{USER_{{ID}^{*}}}\overset{\text{\$}}{\leftarrow }{\mathbb{Z}}_{\phi (N)}^{*}$, and sets *X*_*SEM*_*ID*_*i*___ = *Q*_*ID*_*i*__^*d*_*SEM*_*ID*_*i*___^ as the SEM portion of the user private key and *X*_*USER*_*ID*_*i*___ = *f*_*ID*_*i*__ *Q*_*ID*_*i*__^−*d*_*USER*_*ID*_*i*___^ as the user portion of the user private key. It can be seen that
    (7) XSEMIDi×XUSERIDi  =QIDidSEMIDi×fIDiQIDi−dSEMIDi  =fIDi  =XFULLIDi.
    *M* adds 〈*ID*_*i*_, *Q*_*ID*_*i*__, *f*_*ID*_*i*__, *X*_*FULL*_*ID*_*i*___, *X*_*USER*_*ID*_*i*___, *X*_*SEM*_*ID*_*i*___, *d*_*SEM*_*ID*_*i*___, *d*_*USER*_*ID*_*i*___〉 to *L*_*H*_. *M* returns *Q*_*ID*_*i*__ to *I*.
(3) *SEM-Extract* (*ID*_*i*_). If *ID*_*i*_ = ID* and* USER-Extract* (ID*) has been queried before, *M* aborts. If *ID*_*i*_ = ID*, but* USER-Extract* (ID*) has not been queried before, *M* chooses $d_{{SEM}_{{ID}^{*}}}\overset{\text{\$}}{\leftarrow }{\mathbb{Z}}_{\phi (N)}^{*}$ and sets *X*_*SEM*_ID*__ = *Q*_ID*_^d_*SEM*_ID*__^ as the SEM portion of the user private key, saves it in *L*_*H*_, and returns it to *I*. Otherwise for all other *ID*_*i*_ ≠ ID*, *M* just retrieves *X*_*SEM*_*ID*_*i*___ in *L*_*H*_ and returns it to *I*.
(4) *USER-Extract* (*ID*_*i*_). If *ID*_*i*_ = ID* and* USER-Extract* (ID*) has been queried before, *M* aborts. If *ID*_*i*_ = ID*, but* SEM-Extract* (ID*) has not been queried before, *M* chooses $d_{USER_{{ID}^{*}}}\overset{\text{\$}}{\leftarrow }{\mathbb{Z}}_{\phi (N)}^{*}$ and sets *X*_*USER*_ID*__ = *Q*_ID*_^d_*USER*_ID*__^ as the USER portion of the user private key, saves it in *L*_*H*_, and returns it to *I*. Otherwise for all other *ID*_*i*_ ≠ ID*, *M* just retrieves *X*_*USER*_*ID*_*i*___ in *L*_*H*_ and returns it to *I*.
(5) *Identification* (*ID*_*j*_, *session*). *I* will act as the cheating verifier to learn information from valid conversation transcripts from *M*. If *ID*_*i*_ ≠ ID*  *M* just retrieves *ID*_*i*_'s entry in *L*_*H*_ and runs the identification protocol for either the SEM's interactions, the user's interactions, or both combined as in the protocol.

 Otherwise, for *ID*_*j*_ = ID*, *M* then creates a full valid transcript for each *m*th query by picking $z_{{m,USER}_{{ID}^{*}}},z_{{m,SEM}_{{ID}^{*}}}\overset{\text{\$}}{\leftarrow }{\mathbb{Z}}_{q}^{*}$, and $c_{m,{ID}^{*}}\overset{\text{\$}}{\leftarrow }{\mathbb{Z}}_{q}^{*}$ and sets *Z*_*m*, *FULL*_ID*__ = *z*_*m*, *USER*_ID*__ × *z*_*m*, *SEM*_ID*__. *M* also sets *Y*_*m*, *USER*_ID*__ = *z*_*m*,*USER*_ID*__^e^ *Q*_ID*_^−(*c*_*m*,ID*_/2)^, *Y*_*m*, *SEM*_ID*__ = *z*_*m*, *SEM*_ID*__^e^ *Q*_ID*_^−(*c*_*m*,ID*_/2)^, and *Y*_*m*, *FULL*_ID*__ = *z*_*m*,*FULL*_ID*__^e^ *Q*_ID*_^−*c*_*m*,ID*_^. *M* tags the session with *session* = *m*. If *I* queries
(i)
* SEM-Identification* (ID*, *m*), *M* will return 〈*Y*_*m*, *SEM*_ID*__, *c*_*m*,ID*_, *z*_*m*, *SEM*_ID*__〉;
(ii)
* User-Identification* (ID*, *m*), *M* will return 〈*Y*_*m*, *USER*_ID*__, *c*_*m*,ID*_, *z*_*m*, *USER*_ID*__〉;
(iii)
* Full-Identification* (ID*, *m*), *M* will return 〈*Y*_*m*, *FULL*_ID*__, *c*_*m*,ID*_, *z*_*m*, *FULL*_ID*__〉.
 If no session is specified for the query, *M* just returns the next session in sequence. One can see that* SEM-Identification* (ID*, *m*) and* User-Identification* (ID*, *m*) can be combined to create a full and valid transcript on session *m*:
  (8) Ym,FULLID∗H(ID∗)cm,ID∗  =Ym,SEMID∗Ym,USERID∗QID∗cm,ID∗  =zm,SEMID∗eQID∗−(cm,ID∗/2)zm,USERID∗eQID∗−(cm,ID∗/2)QID∗cm,ID∗  =[zm,SEMID∗zm,USERID∗]eQID∗−cm,ID∗QID∗cm,ID∗  =zm,SEMID∗ezm,USERID∗e  =zm,FULLID∗e.

Eventually, *I* stops Phase 1 and outputs the challenge *ID*, ID* on which it wishes to be challenged on. *M* checks if ID* = *ID*_*l*_ from *L*_*H*_ and aborts if not. Otherwise, *M* runs *I* now as a cheating prover on ID* by obtaining its commitment, *Y*_*FULL*_ID*__, selecting a challenge $c_{1}\overset{\text{\$}}{\leftarrow }{\mathbb{Z}}_{q}^{*}$ and obtaining the response *z*_*FULL*_ID*_,1_ from *I*.  *M* then resets *I* to the step whereby *I* just sent *Y*_*FULL*_ID*__, selects a second challenge $c_{2}\overset{\text{\$}}{\leftarrow }{\mathbb{Z}}_{q}^{*}$, and receives *z*_*FULL*_ID*_,2_ as response. It should hold that *z*_*FULL*_ID*_,1_^*e*^ = *Y*_*FULL*_ID*__ *H*(*ID*)^*c*_1_^ and *z*_*FULL*_ID*_,2_^*e*^ = *Y*_*FULL*_ID*__ *H*(*ID*)^*c*_2_^. One can then obtain (*z*_*FULL*_ID*_,1_/*z*_*FULL*_ID*_,2_)^*e*^ = *H*(*ID*)^*c*_1_−*c*_2_^. Since *e* is a prime and −*e* < *c*_1_ − *c*_2_ < *e*, *GCD*(*e*, *c*_1_ − *c*_2_) = 1. Use the extended Euclidean algorithm to obtain integers *S* and *T* such that *eS* + (*c*_1_ − *c*_2_)*T* = 1. It follows that

(9) H(ID∗)=QID∗eS+(c1−c2)Tmod⁡N=(yfID∗e)eS+(c1−c2)Tmod⁡N=(yfID∗e2S)(yfID∗e(c1−c2)T)mod⁡N=(yfID∗e2S)(zFULLID∗,1zFULLID∗,2)eTmod⁡N=[(yfID∗eS)(zFULLID∗,1zFULLID∗,2)T]emod⁡N.

*M* then calculates the solution to the *RSAI* problem as follows:

(10) yfID∗e=[(yfID∗eS)(zFULLID∗,1zFULLID∗,2)T]emod⁡N,ydfID∗=(yfID∗eS)(zFULLID∗,1zFULLID∗,2)Tmod⁡N,yd=(yfID∗eS)(zFULLID∗,1/zFULLID∗,2)TfID∗mod⁡N,yd=(yfID∗eS−1)(zFULLID∗,1zFULLID∗,2)Tmod⁡N.

It remains to calculate the probability of *M* solving the *RSAI* problem and winning the game. The probability of *M* successfully extracting two valid conversation transcripts from *I* is bounded by (*ε*_*IBI*_ − (1/2^*l*(*k*)^))^2^ as given by the reset lemma [20]:

(11) Pr[M  wins  RSAI]  =Pr[M  computes  gab⋀¬abort]  =Pr[M  computes  gab ∣ ¬abort]Pr[¬abort]εRSAI≥(εSMIBI−12l(k))2Pr[¬abort].

Finally calculate Pr[¬abort]. Let *δ* be the probability that *I* issues both a* SEM-Extract* and a* USER-Extract* query on ID* and that *I* makes a total of *q*_*SMIBI*_ of such queries. The probability of *M* answering all the extraction queries is *δ*_*SMIBI*_^*q*^. In Phase 2, the probability of *M* not aborting is if *I* outputs the challenge identity ID* that was not queried before. This is given by the probability 1 − *δ*. Compiling them, the probability of *M* not aborting is *δ* ^*q*_*SMIBI*_^(1 − *δ*). This probability is maximised at *δ*_*opt*_ = 1 − (1/(*q*_*SMIBI*_ + 1)). Using *δ*_*opt*_, the probability that *M* does not abort is at least 1/(2^*l*(*k*)^(*q*_*SMIBI*_ + 1)) because the value (1 − (1/(*q*_*SMIBI*_ + 1)))^*q*_*SMIBI*_^ approaches 1/*e* for large *q*_*SMIBI*_. Therefore, the advantage of *M*, *ε*_*RSAI*_, and the bound of the simulation are given as follows:

(12) εRSAI≥(εSMIBI−12l(k))21(qSMIBI+1)εRSAI(qSMIBI+1)≥(εSMIBI−12l(k))2εSMIBI≤εRSAI(qSMIBI+1)+12l(k).

### 3.2. Security Analysis: Impersonation under Active/Concurrent Attack

**Theorem 6 .**

The GQ-SM-IBI scheme is (*t*_*SMIBI*_, *q*_*SMIBI*_, *ε*_*SMIBI*_)-secure against impersonation under active/concurrent attacks in the random oracle if the *OMCDH* Problem is (*t*_*OMRSAI*_, *q*_*OMRSAI*_, *ε*_*OMRSAI*_)-hard where

(13) $$\begin{array}{c} \varepsilon_{SMIBI}\leq \sqrt{\varepsilon_{OMRSAI}e\left(q_{SMIBI}+1\right)}+\frac{1}{2^{l(k)}}. \end{array}$$

**Proof**

Assume the GQ-SM-IBI scheme is (*t*_*SMIBI*_, *q*_*SMIBI*_, *ε*_*SMIBI*_)-breakable; then a simulator *M* that (*t*_*OMRSAI*_, *q*_*OMRSAI*_, *ε*_*OMRSAI*_)-breaks the *O* *M* *RSAI* problem can be shown. *M* takes in input (*N*, *e*), is given access to *CHALL* and *RSA* oracles, and runs the impersonator *I* as a subroutine. Any* SEM-Extract, User-Extract, SEM-Identification, User-Identification,* and* Full-Identification* queries are preceded by a* Create-User* query. *M* maintains a list *L*_*H*_ = 〈*ID*_*i*_, *Q*_*ID*_*i*__, *f*_*ID*_*i*__, *X*_*FULL*_*ID*_*i*___, *X*_*SEM*_*ID*_*i*___, *X*_*USER*_*ID*_*i*___, *d*_*SEM*_*ID*_*i*___, *d*_*USER*_*ID*_*i*___〉 which is initially empty. The following shows how *M* simulates the environment and oracle queries for *I*.

(1) *Create-User* (*ID*_*i*_). *M* chooses *l* ∈ {1,…, *q*_*H*_} randomly and lets *ID*_*l*_ = ID* at this point. Whenever *I* makes a *H* query on *ID*_*i*_, consider the following.
  (i) If *i* = *l*, *M* will choose $f_{{ID}^{*}}\overset{\text{\$}}{\leftarrow }{\mathbb{Z}}_{N}^{*}$, queries *CHALL* for *w*_0_, and sets *Q*_ID*_ = *w*_0_ *f*_ID*_^e^ and will return *Q*_ID*_ to *I*. It adds 〈*ID*_*l*_, *Q*_ID*_, *f*_ID*_, *X*_*FULL*_ID*__ = ⊥, *X*_*USER*_ID*__ = ⊥, *X*_*SEM*_ID*__ = ⊥, *d*_*SEM*_ID*__ = ⊥, *d*_*USER*_ID*__ = ⊥〉 to *L*_*H*_.
  (ii) Otherwise, for *i* ≠ *l*, *M* chooses $f_{{ID}_{i}}\overset{\text{\$}}{\leftarrow }{\mathbb{Z}}_{q}^{*}$, sets *Q*_*ID*_*i*__ = *f*_*ID*_*i*__^*e*^, and returns *Q*_*ID*_*i*__ to *I*. *M* also sets *X*_*FULL*_*ID*_*i*___ = *f*_*ID*_*i*__, chooses $d_{USER_{{ID}^{*}}}\overset{\text{\$}}{\leftarrow }{\mathbb{Z}}_{\phi (N)}^{*}$, and sets *X*_*SEM*_*ID*_*i*___ = *Q*_*ID*_*i*__^*d*_*SEM*_*ID*_*i*___^ as the SEM portion of the user private key and *X*_*USER*_*ID*_*i*___ = *f*_*ID*_*i*__ *Q*_*ID*_*i*__^−*d*_*USER*_*ID*_*i*___^ as the user portion of the user private key. It can be seen that
    (14) XSEMIDi×XUSERIDi  =QIDidSEMIDi×fIDiQIDi−dSEMIDi  =fIDi  =XFULLIDi.
    *M* adds 〈*ID*_*i*_, *Q*_*ID*_*i*__, *f*_*ID*_*i*__, *X*_*FULL*_*ID*_*i*___, *X*_*USER*_*ID*_*i*___, *X*_*SEM*_*ID*_*i*___, *d*_*SEM*_*ID*_*i*___, *d*_*USER*_*ID*_*i*___〉 to *L*_*H*_. *M* returns *Q*_*ID*_*i*__ to *I*.
(2) *SEM-Extract* (*ID*_*i*_). If *ID*_*i*_ = ID* and* USER-Extract* (ID*) has been queried before, *M* aborts. If *ID*_*i*_ = ID*, but* USER-Extract* (ID*) has not been queried before, *M* chooses $d_{SEM_{{ID}^{*}}}\overset{\text{\$}}{\leftarrow }{\mathbb{Z}}_{\phi (N)}^{*}$ and sets *X*_*SEM*_ID*__ = *Q*_ID*_^d_*SEM*_ID*__^ as the SEM portion of the user private key, saves it in *L*_*H*_, and returns it to *I*. Otherwise for all other *ID*_*i*_ ≠ ID*, *M* just retrieves *X*_*SEM*_*ID*_*i*___ in *L*_*H*_ and returns it to *I*.
(3) *USER-Extract* (*ID*_*i*_). If *ID*_*i*_ = ID* and* SEM-Extract* (ID*) has been queried before, *M* aborts. If *ID*_*i*_ = ID*, but* SEM-Extract* (ID*) has not been queried before, *M* chooses $d_{USER_{{ID}^{*}}}\overset{\text{\$}}{\leftarrow }{\mathbb{Z}}_{\phi (N)}^{*}$ and sets *X*_*USER*_ID*__ = *Q*_ID*_^d_*USER*_ID*__^ as the USER portion of the user private key, saves it in *L*_*H*_, and returns it to *I*. Otherwise for all other *ID*_*i*_ ≠ ID*, *M* just retrieves *X*_*USER*_*ID*_*i*___ in *L*_*H*_ and returns it to *I*.
(4) *Identification* (*ID*_*i*_, *session*). *I* will act as the cheating verifier to learn information from valid conversation interactions from *M*. If *ID*_*i*_ ≠ ID*  *M* just retrieves *ID*_*i*_'s entry in *L*_*H*_ and runs the identification protocol for either the SEM's interactions, the user's interactions, or both combined as in the protocol.

 Otherwise, for ID* = ID*, *M* then creates a full valid conversation for each *m*th query by querying *CHALL* for *W*_*m*_ and setting *Y*_*FULL*_ID*__ = *W*_*m*_. Additionally, *M* chooses $Y_{m,SEM_{{ID}^{*}}}\overset{\text{\$}}{\leftarrow }{\mathbb{Z}}_{N}^{*}$ and sets *Y*_*m*,*User*_ID*__ = *W*_*m*_/*Y*_*m*,*SEM*_ID*__. Upon receiving *c*_*m*,ID*_ from *I*, *M* will query *RSA*(*W*_*m*_(*W*_0_ *f*_ID*_^e^)^*c*_*m*,ID*_^) to receive *V*_*m*_ = (*W*_*m*_(*W*_0_ *f*_ID*_^e^)^*c*_*m*,ID*_^)^*d*^ and sets *z*_*FULL*_ID*__ = *V*_*m*_. *M* then chooses $z_{m,SEM_{{ID}^{*}}}\overset{\text{\$}}{\leftarrow }{\mathbb{Z}}_{N}^{*}$ and then sets *z*_*m*,*USER*_ID*__ = *V*_*m*_/*z*_*m*,*SEM*_ID*__. *M* tags the session with *session* = *m*.
  (i) If *I* queries* SEM-Identification* (ID*, *m*), *M* will commit *Y*_*m*,*SEM*_ID*__ and respond with *z*_*m*,*SEM*_ID*__ upon receiving *c*_*m*,ID*_.
  (ii) If *I* queries* User-Identification* (ID*, *m*), *M* will commit *Y*_*m*,*USER*_ID*__ and respond with *z*_*m*,*USER*_ID*__ upon receiving *c*_*m*,ID*_.
  (iii) If *I* queries* Full-Identification* (ID*, *m*), *M* will commit *Y*_*m*,*FULL*_ID*__ and respond with *z*_*m*,*FULL*_ID*__ upon receiving *c*_*m*,ID*_.
 If no session is specified for the query, *M* just returns the next session in sequence. One can see that* SEM-Identification* (ID*, *m*) and* User-Identification* (ID*, *m*) can be combined to create a full and valid conversation on session *m*:
  (15) Ym,FULLID∗H(ID∗)cm,ID∗  =Ym,SEMID∗Ym,USERID∗QID∗cm,ID∗  =Ym,SEMID∗WmYm,SEMID∗(W0fID∗e)cm,ID∗  =Wm(W0fID∗e)cm,ID∗  =[Wm(W0fID∗e)cm,ID∗]de  =[[Wm(W0fID∗e)cm,ID∗]d]e  =[Vm]e  =(Vmzm,SEMID∗)e(zm,SEMID∗)e  =zm,SEMID∗ezm,USERID∗e  =zm,FULLID∗e.

Eventually, *I* stops Phase 1 and outputs the challenge *ID*, ID* on which it wishes to be challenged on. *M* checks if ID* = *ID*_*l*_ from *L*_*H*_ and aborts if not. Otherwise, *M* runs *I* now as a cheating prover on ID*. *M* runs by obtaining its commitment, *Y*_*FULL*_ID*__, selects a challenge $c_{1}\overset{\text{\$}}{\leftarrow }{\mathbb{Z}}_{q}^{*}$, and obtains the response *z*_*FULL*_ID*_,1_ from *I*. *M* then resets *I* to the step whereby *I* just sent *Y*_*FULL*_ID*__, selects a second challenge $c_{2}\overset{\text{\$}}{\leftarrow }{\mathbb{Z}}_{q}^{*}$, and receives *z*_*FULL*_ID*_,2_ as response. It should hold that *z*_*FULL*_ID*_,1_^*e*^ = *Y*_*FULL*_ID*__ *H*(*ID*)^*c*_1_^ and *z*_*FULL*_ID*_,2_^*e*^ = *Y*_*FULL*_ID*__ *H*(*ID*)^*c*_2_^. One can then obtain (*z*_*FULL*_ID*_,1_/*z*_*FULL*_ID*_,2_)^*e*^ = *H*(*ID*)^*c*_1_−*c*_2_^. Since *e* is a prime and −*e* < *c*_1_ − *c*_2_ < *e*, *GCD*(*e*, *c*_1_ − *c*_2_) = 1. Use the extended Euclidean algorithm to obtain integers *S* and *T* such that *eS* + (*c*_1_ − *c*_2_)*T* = 1. It follows that

(16) H(ID∗)=QID∗eS+(c1−c2)Tmod⁡N=(w0fID∗e)eS+(c1−c2)Tmod⁡N=(w0fID∗e2S)(w0fID∗e(c1−c2)T)mod⁡N=(w0fID∗e2S)(zFULLID∗,1zFULLID∗,2)eTmod⁡N=[(w0fID∗eS)(zFULLID∗,1zFULLID∗,2)T]emod⁡N.

*M* then calculates the solution to the *RSAI* problem as follows:

(17) $$\begin{array}{c} w_{0}f_{{ID}^{*}}^{e}={\left[\left(w_{0}f_{{ID}^{*}}^{eS}\right){\left(\frac{z_{FULL_{{ID}^{*}},1}}{z_{FULL_{{ID}^{*}},2}}\right)}^{T}\right]}^{e}\mathrm{mod}N,\\ w_{0}^{d}f_{{ID}^{*}}=\left(w_{0}f_{{ID}^{*}}^{eS}\right){\left(\frac{z_{FULL_{{ID}^{*}},1}}{z_{FULL_{{ID}^{*}},2}}\right)}^{T}\mathrm{mod}N,\\ w_{0}^{d}=\frac{\left(w_{0}f_{{ID}^{*}}^{eS}\right){\left(z_{FULL_{{ID}^{*}},1}/z_{FULL_{{ID}^{*}},2}\right)}^{T}}{f_{{ID}^{*}}}\mathrm{mod}N,\\ w_{0}^{d}=\left(w_{0}f_{{ID}^{*}}^{eS-1}\right){\left(\frac{z_{FULL_{{ID}^{*}},1}}{z_{FULL_{{ID}^{*}},2}}\right)}^{T}\mathrm{mod}N. \end{array}$$

*M* proceeds to calculate the solutions to the other challenges as follows:

(18) $$\begin{array}{c} w_{j}=V_{j}{\left(w_{0}f_{{ID}^{*}}\right)}^{-cm,{ID}^{*}}. \end{array}$$

The probability study for the simulation above is similar to that of the impersonation under passive attack game and is therefore omitted.

## 4. BNN-SM-IBI: An DL-Based Security-Mediated IBI Scheme

The BNN-SM-IBI scheme is derived from the BNN-IBI scheme proposed in the work of [4] and is provably secure against passive attackers assuming the DL assumption and against active/concurrent attackers assuming the *OMDL* assumption.

The BNN-SM-IBI scheme is constructed as follows.

1. *Setup* (1^*k*^). It takes in the security parameter 1^*k*^. It randomly selects $x\overset{\text{\$}}{\leftarrow }{\mathbb{Z}}_{q}$, a generator $g\overset{\text{\$}}{\leftarrow }G$ and computes *X* = *g* ^*x*^. Setup also chooses *H* : {0,1}* → *ℤ*_*q*_ and publishes the system parameters *mpk* = 〈*G*, *q*, *g*, *X*, *H*〉. The master private key *msk* = *x* is kept secret.
2. *Extract* (*mpk*, *msk*, *I*D). It takes in *mpk*, *msk* = *s*, and *ID* as input. It calculates *H*(*ID*), picks $r_{FULL},r_{SEM},s_{SEM}\overset{\text{\$}}{\leftarrow }{\mathbb{Z}}_{q}$, calculates *s*_*FULL*_ = *r*_*FULL*_ + *xH*(*ID*) and *R*_*FULL*_ = *g* ^*r*_*FULL*_^, and sets the full user private key as *usk*_*FULL*_ = 〈*s*_*FULL*_, *R*_*FULL*_〉. Additionally, it sets *s*_*USER*_ = *s*_*FULL*_ − *s*_*SEM*_, *r*_*USER*_ = *r*_*FULL*_ − *r*_*SEM*_, *R*_*USER*_ = *g* ^*r*_*USER*_^, *R*_*SEM*_ = *g* ^*r*_*SEM*_^. It then sets *usk*_*USER*_ = 〈*s*_*USER*_, *R*_*USER*_〉 and *usk*_*SEM*_ = 〈*s*_*SEM*_, *R*_*SEM*_〉. It gives *usk*_*USER*_ to the user as its partial private key and *usk*_*SEM*_ to the security mediator as its partial private key and keeps *usk*_*FULL*_ secret.
3. *Identification Protocol. *It is run by the* User-Prover* and* SEM-Prover* with* Verifier* as such.
  - *User-Prover* sends its *ID* to* Sem-Prover*, chooses $y_{USER}\overset{\text{\$}}{\leftarrow }{\mathbb{Z}}_{q}$, and sets *Y*_*USER*_ = *g* ^*y*_*USER*_^ and *S*_*User*_ = *g* ^*s*_*USER*_^.* SEM-Prover* upon receiving *ID* checks if it is still valid and returns error if it is revoked. Otherwise,* SEM-Prover* chooses $y_{SEM}\overset{\text{\$}}{\leftarrow }{\mathbb{Z}}_{q}$ and sends *Y*_*SEM*_ = *g* ^*y*_*SEM*_^, *S*_*SEM*_ = *g* ^*s*_*SEM*_^, and *R*_*SEM*_ to* User-Prover*.* User-Prover* combines *Y*_*FULL*_ = *Y*_*SEM*_ × *Y*_*USER*_, *S*_*FULL*_ = *S*_*SEM*_ × *S*_*USER*_, *R*_*FULL*_ = *R*_*SEM*_ × *R*_*USER*_ and sends *Y*_*FULL*_, *S*_*FULL*_, and *R*_*FULL*_ to* Verifier*.
  - *Verifier* chooses a random challenge $c\overset{\text{\$}}{\leftarrow }{\mathbb{Z}}_{q}$ and sends it to* User-Prover*.* User-Prover* relays *c* to* SEM-Prover*.
  - *SEM-Prover* calculates its half of the response *z*_*SEM*_ = *y*_*SEM*_ + *cs*_*SEM*_ and sends it to* User-Prover*.* User-Prover* combines it with his response *z*_*USER*_ = *y*_*USER*_ + *cs*_*USER*_ to create *z*_*FULL*_ = *z*_*SEM*_ + *z*_*USER*_ and sends it as a response to* Verifier*.

 
*Verifier* checks if *g* ^*z*_*FULL*_^ = *Y*_*FULL*_ *R*_*FULL*_^*c*^ *X* ^*H*(*ID*)*c*^ and accepts if yes; otherwise it outputs reject.

To check for completeness,

(19) gzFULL=gzSEM+zUSER=gySEM+csSEM+yUSER+csUSER=gySEM+yUSER+c(sSEM+sUSER)=gyFULL+c(sFULL)=gyFULL+c(rFULL+xH(ID))=gyFULLgrFULLcgxH(ID)c=YFULLRFULLcXH(ID)c.

### 4.1. Security Analysis: Impersonation under Passive Attack

**Theorem 7 .**

The BNN-SM-IBI scheme is (*t*_*SMIBI*_, *q*_*SMIBI*_, *ε*_*SMIBI*_)-secure against impersonation under passive attacks in the random oracle if the DL problem is (*t*_*DL*_, *ε*_*DL*_)-hard where

(20) $$\begin{array}{c} \varepsilon_{SMIBI}\leq \sqrt{\varepsilon_{DL}e\left(q_{SMIBI}+1\right)}+\frac{1}{q}. \end{array}$$

**Proof**

Assume the BNN-SM-IBI scheme is (*t*_*SMIBI*_, *q*_*SMIBI*_, *ε*_*SMIBI*_)-breakable; then a simulator *M* that (*t*_*DL*_, *ε*_*DL*_)-breaks the DL problem can be shown. *M* takes in input (*g*, *A* = *g* ^*a*^) and runs the impersonator *I* as a subroutine. Without loss of generality, it can be assumed that any* SEM-Extract*,* User-Extract*,* SEM-Identification*,* User-Identification*, and* Full-Identification* queries are preceded by a* Create-User* query. To avoid collision and consistently respond to these queries, *M* maintains a list *L*_*H*_ = 〈*ID*_*i*_, *Q*_*ID*_*i*__, *S*_*FULL*_*ID*_*i*___, *S*_*SEM*_*ID*_*i*___, *S*_*USER*_*ID*_*i*___, *s*_*FULL*_*ID*_*i*___, *s*_*SEM*_*ID*_*i*___, *s*_*USER*_*ID*_*i*___, *r*_*FULL*_*ID*_*i*___, *r*_*SEM*_*ID*_*i*___, *r*_*USER*_*ID*_*i*___, *R*_*FULL*_*ID*_*i*___, *R*_*SEM*_*ID*_*i*___, *R*_*USER*_*ID*_*i*___〉 which is initially empty. The following shows how *M* simulates the environment and oracle queries for *I*.

1. *Setup*. *M* sets the system parameters as *mpk* = 〈*G*, *q*, *g*, *X* = *g* ^*x*^, *H*〉 and keeps master private key *x* secret. It sends *mpk* to *I*.
2. *Create-User *(*ID*_*i*_). *M* chooses *l* ∈ {1,…, *q*_*H*_} randomly and lets *ID*_*l*_ = ID* at this point. Whenever *I* makes a *H* query on *ID*_*i*_, consider the following.
  - If *i* = *l*, *M* chooses $Q_{{I\text{D}}^{*}}\overset{\text{\$}}{\leftarrow }{\mathbb{Z}}_{q}$ and sets *S*_*FULL*_*I*D*__ = *A* and *R*_*FULL*_ID*__ = *AX* ^*Q*_ID*_^ and returns *Q*_ID*_ to *I*. *M* adds 〈*ID*_*l*_, *Q*_ID*_, *S*_*FULL*_ID*__, *S*_*SEM*_ID*__ = ⊥, *S*_*USER*_ID*__, *s*_*FULL*_ID*__ = ⊥, *s*_*SEM*_ID*__, *s*_*USER*_ID*__ = ⊥, *r*_*FULL*_ID*__ = ⊥, *r*_*SEM*_ID*__ = ⊥, *r*_*USER*_ID*__ = ⊥, *R*_*FULL*_ID*__, *R*_*SEM*_ID*__ = ⊥, *R*_*USER*_ID*__ = ⊥〉 to *L*_*H*_.
  - Otherwise, for *i* ≠ *l*, *M* chooses $Q_{{ID}_{i}}\overset{\text{\$}}{\leftarrow }{\mathbb{Z}}_{q}^{*}$ and returns *Q*_*ID*_*i*__. It then chooses $r_{FULL_{{ID}_{i}}},r_{SEM_{{ID}_{i}}},s_{SEM_{{ID}_{i}}}\overset{\text{\$}}{\leftarrow }{\mathbb{Z}}_{q}^{*}$, calculates *s*_*FULL*_*ID*_*i*___ = *r*_*FULL*_*ID*_*i*___ + *xQ*, and sets *r*_*USER*_*ID*_*i*___ = *r*_*FULL*_*ID*_*i*___ − *r*_*SEM*_*ID*_*i*___ and *s*_*USER*_*ID*_*i*___ = *s*_*FULL*_*ID*_*i*___ − *s*_*SEM*_*ID*_*i*___. *M* adds 〈*ID*_*i*_, *Q*_*ID*_*i*__, *S*_*FULL*_*ID*_*i*___, *S*_*SEM*_*ID*_*i*___, *S*_*USER*_*ID*_*i*___, *s*_*FULL*_*ID*_*i*___, *s*_*SEM*_*ID*_*i*___, *s*_*USER*_*ID*_*i*___, *r*_*FULL*_*ID*_*i*___, *r*_*SEM*_*ID*_*i*___, *r*_*USER*_*ID*_*i*___, *R*_*FULL*_*ID*_*i*___, *R*_*SEM*_*ID*_*i*___, *R*_*USER*_*ID*_*i*___〉 to *L*_*H*_.
3. *SEM-Extract* (*ID*_*i*_). If *ID*_*i*_ = ID* and* USER-Extract* (ID*) has been queried before, *M* aborts. If *ID*_*i*_ = ID*, but* USER-Extract* (ID*) has not been queried before, *M* will choose $r_{SEM_{{ID}^{*}}},s_{SEM_{{ID}^{*}}}\overset{\text{\$}}{\leftarrow }{\mathbb{Z}}_{q}$ and sets *R*_*SEM*_ID*__ = *g* ^*r*_*SEM*_ID*__^ and *S*_*SEM*_ID*__ = *g* ^*s*_*SEM*_ID*__^. *M* also sets *S*_*USER*_ID*__ = *S*_*FULL*_ID*__/*S*_*SEM*_ID*__ and *R*_*USER*_ID*__ = *R*_*FULL*_ID*__/*R*_*SEM*_ID*__, saves these values in *L*_*H*_, and returns them to *I*. These values will be used in the event of an* Identification* query later on. Otherwise for all other *ID*_*i*_ ≠ ID*, *M* just retrieves 〈*s*_*SEM*_*ID*_*i*___, *R*_*SEM*_*ID*_*i*___〉 in *L*_*H*_ and returns it to *I*.
4. *USER-Extract* (*ID*_*i*_). If *ID*_*i*_ = ID* and* SEM-Extract* (ID*) has been queried before, *M* aborts. If *ID*_*i*_ = ID*, but* SEM-Extract* (ID*) has not been queried before, *M* will choose $r_{USER_{{ID}^{*}}},s_{USER_{{ID}^{*}}}\overset{\text{\$}}{\leftarrow }{\mathbb{Z}}_{q}$ and sets *R*_*USER*_ID*__ = *g* ^*r*_*USER*_ID*__^ and *S*_*USER*_ID*__ = *g* ^*s*_*USER*_ID*__^. *M* also sets *S*_*SEM*_ID*__ = *S*_*FULL*_ID*__/*S*_*USER*_ID*__ and *R*_*SEM*_ID*__ = *R*_*FULL*_ID*__/*R*_*USER*_ID*__, saves these values in *L*_*H*_, and returns them to *I*. These values will be used in the event of an* Identification* query later on. Otherwise for all other *ID*_*i*_ ≠ ID*, *M* just retrieves 〈*s*_*USER*_*ID*_*i*___, *R*_*USER*_*ID*_*i*___〉 in *L*_*H*_ and returns it to *I*.
5. *Identification* (*ID*_*j*_, *session*). *I* will act as the cheating verifier to learn information from valid conversation transcripts from *M*. If *ID*_*j*_ ≠ ID*, *M* just retrieves *ID*_*j*_'s entry in *L*_*H*_ and runs the identification protocol for either the SEM's interactions, the user's interactions, or both combined as in the protocol.

 Otherwise, for *ID*_*j*_ = ID*, *M* then creates a full valid transcript for each *m*th query by picking $z_{m,FULL_{{ID}^{*}}},z_{m,SEM_{{ID}^{*}}},c_{m,{ID}^{*}}\overset{\text{\$}}{\leftarrow }{\mathbb{Z}}_{q}$. *M* retrieves *S*_*FULL*_ID*__, *R*_*FULL*_ID*__ and sets *Y*_*m*,*FULL*_ID*__ = *g* ^*z*_*m*,*FULL*_ID*__^ *S*_*FULL*_ID*__^−*c*_*m*,ID*_^. Additionally, *M* chooses $z_{m,SEM_{{ID}^{*}}}\overset{\text{\$}}{\leftarrow }{\mathbb{Z}}_{q}$, calculates *z*_*m*,*USER*_ID*__ = *z*_*m*,*FULL*_ID*__ − *z*_*m*,*SEM*_ID*__, and retrieves *S*_*SEM*_ID*__, *R*_*SEM*_ID*__ and *S*_*USER*_ID*__, *R*_*USER*_ID*__ from *L*_*H*_ as well. *M* then sets *Y*_*m*,*SEM*_ID*__ = *g* ^*z*_*m*,*SEM*_ID*__^ *S*_*SEM*_ID*__^c_*m*,ID*_^ and *Y*_*m*,*USER*_ID*__ = *g* ^*z*_*m*,*USER*_ID*__^ *S*_*USER*_ID*__^c_*m*,ID*_^. *M* tags the session with *session* = *m*. If *I* queries
  (i) * SEM-Identification* (ID*, *m*), *M* will return 〈*Y*_*m*,*SEM*_ID*__, *S*_*SEM*_ID*__, *R*_*SEM*_ID*__, *c*_*m*,ID*_, *z*_*m*,*SEM*_ID*__〉;
  (ii) * User-Identification* (ID*, *m*), *M* will return 〈*Y*_*m*,*USER*_ID*__, *S*_*USER*_ID*__, *R*_*USER*_ID*__, *c*_*m*,ID*_ *z*_*m*,*USER*_ID*__〉;
  (iii) Full-Identification (ID*, *m*), *M* will return 〈*Y*_*m*,*FULL*_ID*__, *S*_*FULL*_ID*__, *R*_*FULL*_ID*__, *c*_*m*,ID*_ *z*_*m*,*FULL*_ID*__〉.
 If no session is specified for the query, *M* just returns the next session in sequence. One can see that* SEM-Identification*  (ID*, *m*) and* User-Identification*  (ID*, *m*) can be combined to create a full and valid transcript on session *m*:
  (21) Ym,FULLID∗RFULLID∗cm,ID∗XH(ID∗)cm,ID∗ =Ym,USERID∗Ym,SEMID∗RUSERID∗cm,ID∗RSEMID∗cm,ID∗  ×XH(ID∗)cm,ID∗ =(gzm,USERID∗SUSERID∗−cm,ID∗gzm,SEMID∗)SSEMID∗−cm,ID∗  ×(RUSERID∗RSEMID∗)cm,ID∗XH(ID∗)cm,ID∗ =gzm,USERID∗gzm,SEMID∗(SUSERID∗SSEMID∗)−cm,ID∗  ×(RUSERID∗RSEMID∗)cm,ID∗XH(ID∗)cm,ID∗ =gzm,USERID∗gzm,SEMID∗(SFULLID∗SSEMID∗SSEMID∗)−cm,ID∗  ×(RFULLID∗RSEMID∗RSEMID∗)cm,ID∗XH(ID∗)cm,ID∗ =gzm,FULLID∗A−cm,ID∗Acm,ID∗X−H(ID∗)cm,ID∗  ×XH(ID∗)cm,ID∗ =gzm,FULLID∗.

Eventually, *I* stops Phase 1 and outputs the challenge *ID*, ID* on which it wishes to be challenged on. *M* checks if *ID* = *ID*_*l*_ from *L*_*H*_ and aborts if not. Otherwise, *M* runs *I* now as a cheating prover on ID* by obtaining its commitment, *Y*_*FULL*_ID*__, *S*_*FULL*_ID*__, *R*_*FULL*_ID*__ selecting a challenge $c_{1}\overset{\text{\$}}{\leftarrow }{\mathbb{Z}}_{q}^{*}$ and obtaining the response *z*_*FULL*_ID*_,1_ from *I*. *M* then resets *I* to the step whereby *I* just sent *Y*_*FULL*_ID*__, *S*_*FULL*_ID*__, *R*_*FULL*_ID*__, selects a second challenge $c_{2}\overset{\text{\$}}{\leftarrow }{\mathbb{Z}}_{q}^{*}$, and receives *z*_*FULL*_ID*_,2_ as response. *M* is then able to extract the full user private key as follows:

(22) zFULLID∗,1−zFULLID∗,2c1−c2  =yFULLID∗+c1a−yFULLID∗+c2ac1−c2  =(c1−c2)ac1−c2  =a.

*M* outputs *a* as the discrete log solution.

It remains to calculate the probability of *M* solving the CDH problem and winning the game. The probability of *M* successfully extracting two valid conversation transcripts from *I* is bounded by (*ε*_*SMIBI*_ − (1/*q*))^2^ as given by the reset lemma [20]:

(23) Pr[M  wins  DL]  =Pr[M  computes  a  ⋀¬abort]  =Pr[M  computes  a ∣ ¬abort]Pr[¬abort]εDL≥(εSMIBI−1q)2Pr[¬abort].

Finally, let *δ* be the probability that *I* issues a* SEM-Extract* and a* USER-Extract* query on ID* and that *I* makes a total of *q*_*SMIBI*_ of such queries. The probability of *M* answering all the extraction queries is *δ*_*I*_^*q*^. In Phase 2, the probability of *M* not aborting is if *I* outputs the challenge identity ID* that was not queried before. This is given by the probability 1 − *δ*. Compiling them, the probability of *M* not aborting is *δ* ^*q*_*SMIBI*_^(1 − *δ*). This value is maximised at *δ*_*opt*_ = 1 − (1/(*q*_*SMIBI*_ + 1)). Using *δ*_*opt*_, The probability that *M* does not abort is at least 1/*e*(*q*_*SMIBI*_ + 1) because the value (1 − (1/(*q*_*SMIBI*_ + 1)))^*q*_*SMIBI*_^ approaches 1/*e* for large *q*_*SMIBI*_. Therefore, the advantage of *M*, *ε*_DL_, and the bound of the simulation is given as follows:

(24) εDL≥(εSMIBI−1q)21e(qSMIBI+1)εDLe(qSMIBI+1)≥(εSMIBI−1q)2εSMIBI≤εDLe(qSMIBI+1)+1q.

### 4.2. Security Analysis: Impersonation under Active/Concurrent Attack

**Theorem 8 .**

The BNN-SM-IBI scheme is (*t*_*SMIBI*_, *q*_*SMIBI*_, *ε*_*SMIBI*_)-secure against impersonation under active/concurrent attacks in the random oracle if the *OMCDH* Problem is (*t*_*OMCDH*_, *q*_*OMCDH*_, *ε*_*OMCDH*_)-hard where

(25) $$\begin{array}{c} \varepsilon_{SMIBI}\leq \sqrt{\varepsilon_{OMCDH}e\left(q_{SMIBI}+1\right)}+\frac{1}{q}. \end{array}$$

**Proof**

Assume the BNN-SM-IBI scheme is (*t*_*SMIBI*_, *q*_*SMIBI*_, *ε*_*SMIBI*_)-breakable; then a simulator *M* that (*t*_*OMCDH*_, *q*_*OMCDH*_, *ε*_*OMCDH*_)-breaks the *OMCDH* Problem can be shown. *M* takes in input (*g*, *g* ^*a*^), is given access to *CHALL* and *DLOG* oracles, and runs the impersonator *I* as a subroutine. Without loss of generality, it can be assumed that any* SEM-Extract*,* User-Extract*,* SEM-Identification*,* User-Identification,* and* Full-Identification* queries are preceded by a* Create-User* query. To avoid collision and consistently respond to these queries, *M* maintains a list *L*_*H*_ = 〈*ID*_*i*_, *Q*_*ID*_*i*__, *S*_*FULL*_*ID*_*i*___, *S*_*SEM*_*ID*_*i*___, *S*_*USER*_*ID*_*i*___, *s*_*FULL*_*ID*_*i*___, *s*_*SEM*_*ID*_*i*___, *s*_*USER*_*ID*_*i*___, *r*_*FULL*_*ID*_*i*___, *r*_*SEM*_*ID*_*i*___, *r*_*USER*_*ID*_*i*___, *R*_*FULL*_*ID*_*i*___, *R*_*SEM*_*ID*_*i*___, *R*_*USER*_*ID*_*i*___〉 which is initially empty. The following shows how *M* simulates the environment and oracle queries for *I*.

1. *Setup*. *M* sets the system parameters as mpk  = 〈*G*, *q*, *g*, *X* = *g* ^*x*^, *H*〉 and keeps master private key *x* secret. It sends mpk to *I*.
2. *Create-User* (*ID*_*i*_). *M* chooses *l* ∈ {1,…, *q*_*H*_} randomly and lets *ID*_*l*_ = ID* at this point. Whenever *I* makes a *H* query on *ID*_*i*_, consider the following.
  - If *i* = *l*, *M* chooses $Q_{{ID}^{*}}\overset{\text{\$}}{\leftarrow }{\mathbb{Z}}_{q}$, queries *CHALL* for *W*_0_, and sets *S*_*FULL*_ID*__ = *W*_0_ and *R*_*FULL*_ID*__ = *W*_0_ *X* ^*Q*_ID*_^. *M* returns *Q*_ID*_ to *I*. *M* adds *L*_*H*_ = 〈*ID*_*l*_, *Q*_ID*_, *S*_*FULL*_ID*__, *S*_*SEM*_ID*__ = ⊥, *S*_*USER*_ID*__ = ⊥, *s*_*FULL*_ID*__ = ⊥, *s*_*SEM*_ID*__ = ⊥, *s*_*USER*_ID*__ = ⊥, *r*_*FULL*_ID*__ = ⊥, *r*_*SEM*_ID*__ = ⊥, *r*_*USER*_ID*__ = ⊥, *R*_*FULL*_ID*__, *R*_*SEM*_ID*__ = ⊥, *R*_*USER*_ID*__ = ⊥〉 to *L*_*H*_.
  - Otherwise, for *i* ≠ *l*, *M* chooses $Q_{{ID}_{i}}\overset{\text{\$}}{\leftarrow }{\mathbb{Z}}_{q}^{*}$ and returns *Q*_*ID*_*i*__. It then chooses $r_{FULL_{{ID}_{i}}},r_{SEM_{{ID}_{i}}},s_{SEM_{{ID}_{i}}}\overset{\text{\$}}{\leftarrow }{\mathbb{Z}}_{q}^{*}$, calculates *s*_*FULL*_*ID*_*i*___ = *r*_*FULL*_*ID*_*i*___ + *xQ*, and sets *r*_*USER*_*ID*_*i*___ = *r*_*FULL*_*ID*_*i*___ − *r*_*SEM*_*ID*_*i*___ and *s*_*USER*_*ID*_*i*___ = *s*_*FULL*_*ID*_*i*___ − *s*_*SEM*_*ID*_*i*___. *M* adds *L*_*H*_ = 〈*ID*_*i*_, *Q*_*ID*_*i*__, *S*_*FULL*_*ID*_*i*___, *S*_*SEM*_*ID*_*i*___, *S*_*USER*_*ID*_*i*___, *s*_*FULL*_*ID*_*i*___, *s*_*SEM*_*ID*_*i*___, *s*_*USER*_*ID*_*i*___, *r*_*FULL*_*ID*_*i*___, *r*_*SEM*_*ID*_*i*___, *r*_*USER*_*ID*_*i*___, *R*_*FULL*_*ID*_*i*___, *R*_*SEM*_*ID*_*i*___, *R*_*USER*_*ID*_*i*___〉 to *L*_*H*_.
3. *SEM-Extract* (*ID*_*i*_). If *ID*_*i*_ = ID* and* USER-Extract* (ID*) has been queried before, *M* aborts. If *ID*_*i*_ = ID*, but* USER-Extract* (ID*) has not been queried before, *M* will choose $r_{S\text{E}M_{{ID}^{*}}},s_{SEM_{{ID}^{*}}}\overset{\text{\$}}{\leftarrow }{\mathbb{Z}}_{q}$ and sets *R*_*SEM*_ID*__ = *g* ^*r*_*SEM*_ID*__^ and *S*_*SEM*_ID*__ = *g* ^*s*_*SEM*_ID*__^. *M* also sets *S*_*USER*_ID*__ = *S*_*FULL*_ID*__/*S*_*SEM*_ID*__ and *R*_*USER*_ID*__ = *R*_*FULL*_ID*__/*R*_*SEM*_ID*__, saves these values in *L*_*H*_, and returns them to *I*. These values will be used in the event of an* Identification* query later on. Otherwise for all other *ID*_*i*_ ≠ ID*, *M* just retrieves 〈*s*_*SEM*_*ID*_*i*___, *R*_*SEM*_*ID*_*i*___〉 in *L*_*H*_ and returns it to *I*.
4. *USER-Extract* (*ID*_*i*_). If *ID*_*i*_ = ID* and* SEM-Extract* (ID*) has been queried before, *M* aborts. If *ID*_*i*_ = ID*, but* SEM-Extract* (ID*) has not been queried before, *M* will choose $r_{USER_{{ID}^{*}}},s_{USER_{{ID}^{*}}}\overset{\text{\$}}{\leftarrow }{\mathbb{Z}}_{q}$ and sets *R*_*USER*_ID*__ = *g* ^*r*_*USER*_ID*__^ and *S*_*USER*_ID*__ = *g* ^*s*_*USER*_ID*__^. *M* also sets *S*_*SEM*_ID*__ = *S*_*FULL*_ID*__/*S*_*USER*_ID*__ and *R*_*SEM*_ID*__ = *R*_*FULL*_ID*__/*R*_*USER*_ID*__, saves these values in *L*_*H*_, and returns them to *I*. These values will be used in the event of an Identification query later on. Otherwise for all other *ID*_*i*_ ≠ ID*, *M* just retrieves 〈*s*_*USER*_*ID*_*i*___, *R*_*USER*_*ID*_*i*___〉 in *L*_*H*_ and returns it to *I*.
5. *Identification* (*ID*_*j*_, *session*). *I* will act as the cheating verifier to learn information from valid conversations from *M*. If *ID*_*j*_ ≠ ID*, *M* just retrieves *ID*_*j*_'s entry in *L*_*H*_ and runs the identification protocol for either the SEM's interactions, the user's interactions, or both combined as in the protocol.

 Otherwise, for *ID*_*i*_ = ID*, *M* then creates a full valid conversation for each *m*th query by querying *CHALL* for *W*_*m*_ and setting *Y*_*m*,*FULL*_ID*__ = *W*_*m*_. *M* also retrieves. Additionally, *M* chooses $Y_{m,SEM_{{ID}_{j}}}\overset{\text{\$}}{\leftarrow }G$ and sets *Y*_*m*,*User*_*ID*_*j*___ = *W*_*m*_/*Y*_*m*,*SEM*_*ID*_*j*___. *M* also retrieves *S*_*m*,*FULL*_*ID*_*j*___, *S*_*m*,*SEM*_*ID*_*j*___, *S*_*m*,*USER*_*ID*_*j*___, *R*_*m*,*FULL*_*ID*_*j*___, *R*_*m*,*SEM*_*ID*_*j*___, and *R*_*m*,*USER*_*ID*_*j*___ from *L*_*H*_. Upon receiving *c*_*m*,*ID*_*j*__ from *I*, *M* will query *z*_*m*,*FULL*_*ID*_*j*___ = *DLOG*(*W*_*m*_ *W*_0_^*c*_*m*,*ID*_*j*__^), selects $z_{m,SEM_{{ID}_{j}}}\overset{\text{\$}}{\leftarrow }{\mathbb{Z}}_{q}$, and sets *z*_*m*,*USER*_*ID*_*j*___ = *z*_*m*,*FULL*_*ID*_*j*___ − *z*_*m*,*SEM*_*ID*_*j*___. *M* tags the session with *session* = *m*.
  (a) If *I* queries* SEM-Identification* (*ID*_*j*_, *m*), *M* will commit *Y*_*m*,*SEM*_*ID*_*j*___, *S*_*SEM*_*ID*_*j*___, *R*_*SEM*_*ID*_*j*___ and respond with *z*_*m*,*SEM*_*ID*_*j*___ upon receiving *c*_*m*,*ID*_*j*__.
  (b) If *I* queries* User-Identification* (*ID*_*j*_, *m*), *M* will commit *Y*_*m*,*USER*_*ID*_*j*___, *S*_*USER*_*ID*_*j*___, *R*_*USER*_*ID*_*j*___ and respond with *z*_*m*,*USER*_*ID*_*j*___ upon receiving *c*_*m*,*ID*_*j*__.
  (c) If *I* queries* Full-Identification* (*ID*_*j*_,*m*), M will commit *Y*_*m*,*FULL*_*ID*_*j*___, *S*_*FULL*_*ID*_*j*___, *R*_*FULL*_*ID*_*j*___ and respond with *z*_*m*,*FULL*_*ID*_*j*___ upon receiving *c*_*m*,*ID*_*j*__.
 If no session is specified for the query, *M* just returns the next session in sequence. One can see that* SEM-Identification* (*ID*_*j*_, *m*) and* User-Identification* (ID*, *m*) can be combined to create a full and valid conversation on session *m*:
  (26) Ym,FULLIDjRFULLIDjcm,IDjXH(IDj)cm,IDj  =Ym,USERIDjYm,SEMIDjRUSERIDjcm,IDjRSEMIDjcm,IDjXH(IDj)cm,IDj  =WmYm,SEMIDjYm,SEMIDj(W0X−H(ID)RSEMIDj)cm,IDj   ×RSEMIDjcm,IDjXH(IDj)cm,IDj  =Wm(W0cm,IDj)X−H(ID)cm,IDjXH(IDj)cm,IDj  =Wm(W0cm,IDj)  =gDLOG(Wm(W0cm,IDj)  =gzm,FULLIDj.

Eventually, *I* stops Phase 1 and outputs the challenge *ID*, ID* on which it wishes to be challenged on. *M* checks if ID* = *ID*_*l*_ from *L*_*H*_ and aborts if not. Otherwise, *M* runs *I* now as a cheating prover on ID*. *M* runs by obtaining its commitment, *Y*_*FULL*_ID*__, *S*_*FULL*_ID*__, *R*_*FULL*_ID*__, selects a challenge $c_{1}\overset{\text{\$}}{\leftarrow }{\mathbb{Z}}_{q}^{*}$, and obtains the response *z*_*FULL*_ID*_,1_ from *I*. *M* then resets *I* to the step whereby *I* just sent *Y*_*FULL*_ID*__, *S*_*FULL*_ID*__, *R*_*FULL*_ID*__, selects a second challenge $c_{2}\overset{\text{\$}}{\leftarrow }{\mathbb{Z}}_{q}^{*}$, and receives *z*_*FULL*_ID*_,2_ as response. *M* is then able to extract the full user private key as follows:

(27) zFULLID∗,1−zFULLID∗,2c1−c2  =yFULLID∗+c1w0−yFULLID∗+c2w0c1−c2  =(c1−c2)w0c1−c2  =w0.

*M* outputs *w*_0_ as the initial discrete log challenge solution.

*M* proceeds to calculate the solutions to the other challenges as follows:

(28) zm,FULLIDj−w0cm,IDj  =DLOG(WmW0cm,IDj)−w0cm,IDj  =[wm+w0cm,IDj]−w0cm,IDj  =wm.

The probability study for the simulation above is similar to that of the impersonation under passive attack game and is therefore omitted.

## 5. Efficiency Analysis

In this section we provide the breakdown of operation costs for both the GQ-SM-IBI scheme and the BNN-SM-IBI scheme.

We measure the operation costs of the GQ-SM-IBI scheme in terms of addition operations in *ℤ*_*ϕ*(*N*)_, multiplication operations in *ℤ*_*ϕ*(*N*)_ and *ℤ*_N_*, and exponentiation operations in *ℤ*_N_*. Overall, the operational costs of the GQ-SM-IBI scheme are given in Table 1.

We also provide the communication costs of the GQ-SM-IBI scheme in Table 2.

As for the BNN-SM-IBI scheme, we measure the operational costs in terms of group operational costs of addition modulo *q*, multiplication modulo *q*, multiplication in group *G*, and exponentiations modulo *q*. Overall, the operational costs of the BNN-SM-IBI scheme are given in Table 3.

We also provide the communication costs of the BNN-SM-IBI scheme in Table 4.

However, it is difficult to compare all these operational costs since the schemes are using operations defined in different fields and groups. Therefore, we built a simulator to generate running time results using a common platform.

We compare the running time of only the identification protocol, since the protocol is invoked every time a prover wishes to perform an interaction with a verifier. We compare the running times of the GQ-SM-IBI scheme and BNN-SM-IBI scheme as well as the original pairing-based scheme by [19]. We name the scheme from [19] as BLS-SM-IBI since its design follows the BLS signature scheme by Boneh et al. from [22]. For GQ-SM-IBI and BNN-SM-IBI the level of security used was 1024-bits, 2048-bits, and 3072-bits. We also compared the results with the equivalent level of security for BLS-SM-IBI pairing-based scheme of 512-bits, 1024-bits, and 1536-bits, respectively.

The simulation was run on a i7-2630QM platform with 4 GB RAM running 64-bit Windows 7. The library used was Java Cryptography Extension. We ran the simulation 100 iterations for each algorithm and took the average running time, measured in nanoseconds as presented in Table 5.

From the results, one can see that the GQ-SM-IBI scheme has the fastest identification protocol running time with the BNN-SM-IBI scheme trailing behind. However, both pairing-free schemes vastly outperform the BLS-SM-IBI that is pairing-based with at least an approximate factor of 6 to 7 for all three security levels. Therefore we obtain faster SM-IBI schemes from pairing-free alternatives.

## 6. Conclusion

We proposed two SM-IBI schemes that have an instant revocation feature and are very efficient. Our schemes outperform the only pairing-based SM-IBI currently known and are provably secure in the random oracle model against both passive and active/concurrent attackers.

## Figures and Tables

**Table 1:** Operation costs for the GQ-SM-IBI scheme.

Algorithm	*A *	*M*1	*M*2	*E *
Setup	0	0	0	0
Extract	1	0	0	3
SEM-Prove	0	0	1	2
User-Prove	0	0	3	2
Verify	0	0	1	2

*A*: addition in *ℤ*_ϕ(*N*)_, *M*1: multiplication in *ℤ*_ϕ(*N*)_, *M*2: multiplication in *ℤ*_N_*, and *E*: exponentiation in *ℤ*_N_*.

**Table 2:** Communication costs between algorithms for the GQ-SM-IBI scheme.

Algorithms	Bitstring |ID|	Elements of |*e* − 1|	Elements in *ℤ*_N_*
TA-User (Extract)	1	0	1
TA-SEM (Extract)	1	0	1
SEM-User (Identification)	1	1	2
User-Verifier (Identification)	1	1	2

**Table 3:** Operation costs for the BNN-SM-IBI scheme.

Algorithm	*A *	*M *	GM	*E *
Setup	0	0	0	1
Extract	3	1	0	3
SEM-Prover	1	1	0	2
User-Prover	2	1	3	2
Verifier	0	1	2	3

*H*: hash operation, *A*: addition mod *q*, *M*: multiplication mod *q*, GM: group multiplication, and *E*: exponentiation mod *q*.

**Table 4:** Communication costs between algorithms for the BNN-SM-IBI scheme.

Algorithms	Bitstring	Elements in *G*_1_	Elements in *ℤ*_*q*_
TA-User (Extract)	1	1	1
TA-SEM (Extract)	1	1	1
SEM-User (Identification)	1	3	2
User-Verifier (Identification)	1	3	2

**Table 5:** Comparison of average simulated runtimes for SM-IBI schemes.

	Identification (ns)
GQ-SM-IBI (1024-bits)	2,158,747
BNN-SM-IBI (1024-bits)	14,064,652
BLS-SM-IBI (512-bits)	104,664,826

GQ-SM-IBI (2048-bits)	7,706,801
BNN-SM-IBI (2048-bits)	82,456,452
BLS-SM-IBI (1024-bits)	487,682,172

GQ-SM-IBI (3072-bits)	17,027,899
BNN-SM-IBI (3072-bits)	182,153,886
BLS-SM-IBI (1536-bits)	1,188,023,987
